# The National Pension Fund

**Published:** 1887-07-23

**Authors:** 


### THE NATIONAL PENSION FUND.

It will save a good deal of trouble if nurses and other hospital officials who propose joining the National Pension Fund will send their names and other particulars to *the Secretary of The Hospitals Association*, Norfolk House, Norfolk Street, W.C., and not to the Editor of The Hospital. Names continue to arrive in considerable numbers, but if those interested desire the Fund to be started before the end of the Jubilee year they must influence and interest all their friends immediately.

### From the Lady Superintendent of Leeds Trained Nurses' Institution.

I have not answered your letter of April 16th, respecting the National Pension Fund for Nurses, as I find nurses are disinclined to enter their names on a register until some rules are made. When they are, I have no doubt that almost all the nurses here will be glad to join. They ask the following questions:—At what age they must first enter their names to receive £25 a year at fifty? What sum they must give per year? In case of their breaking down in health before fifty years of age, whether they could receive any help from the Fund? Being unable to work, whether they could withdraw their money from the Fund? Whether, in case of their death, their relatives could receive any or all the money they had paid in?

These are questions only rules can answer, and until some are made I do not think we shall be able to make much progress. My assistant, Miss ——, wishes me to say she should like her name entered on your register. You will require some capital: do you not consider every hospital or institution that has a certain number of nurses on the register wishing to benefit by the Fund, should be asked to contribute to the capital a certain proportion? When rules are made, I will do my best to help the scheme forward with my influence.

### From a Nurse, Windsor Royal Infirmary.

I cannot tell you how pleased I am to know about the National Pension Fund for Hospital Officials and Nurses. I have long felt the need of something of the kind, and feel sure that when generally known very few, if any, nurses will refuse to join, especially if it included a weekly sum in case of sickness, for nearly all nurses are working for the support of some one dear to them, therefore cannot save for sickness and old age. I think nurses of one year's training should be eligible, for many nurses may have been trained, say, twelve years ago, for twelve months. Well, twelve or even ten years ago, training was not considered so essential as now. Please will you put the names of my two fellow-nurses and my own on your register?

I think it would have been a grand idea if some one had proposed as a Women's Jubilee Memorial to build a Convalescent Home, or House of Rest for Nurses who were unable to work, or required rest and change. I feel sure every nurse would have given what she could spare towards it.

Also I should like to say I like the novel in The Hospital very much, but agree with Miss Twining it would be as well if the scenes were in some other place than a hospital, though they might introduce a doctor in them, as Wilkie Collins does, for instance. I like his works for that reason. Wishing every success to the National Pension Fund and The Hospital generally, for it is a book that was greatly needed.

### From a Lady Member of the Committee of the Blackheath and Charlton Cottage Hospital.

I am much obliged to you for sending the numbers of The Hospital referring to the establishment of the National Pension Fund. I should indeed be glad to see such a Fund set on foot, but can hardly claim the right of being anything but a friend to the proposed scheme. I am neither a nurse nor an official, but for seven years have been very intimately connected with the Blackheath and Charlton Cottage Hospital. I shall be specially interested in noticing what proposal is made for the benefit of nurses who at the present moment may be over 40 years of age. I imagine a larger annual subscription to the Pension Fund would be required from them than from the younger nurses. I know a most capable nurse above the age I mention, who has asked me to make inquiries in this matter; but I presume the subject will be touched upon in later editions, when further details have been under discussion.

